# Internal Transcribed Spacer rDNA and *TEF-1α* Gene Sequencing of Pathogenic Dermatophyte Species and Differentiation of Closely Related Species Using PCR-RFLP of The Topoisomerase II

**DOI:** 10.22074/cellj.2020.6372

**Published:** 2019-09-08

**Authors:** Zahra Salehi, Masoomeh Shams-Ghahfarokhi, Mehdi Razzaghi-Abyaneh

**Affiliations:** 1 Department of Mycology, Faculty of Medical Sciences, Tarbiat Modares University, Tehran, Iran; 2 Department of Mycology, Pasteur Institute of Iran, Tehran, Iran

**Keywords:** Dermatophytes, Gene Sequencing, Polymerase Chain Reaction-Restriction Fragment Length Polymorphism, Topoisomerase II

## Abstract

**Objective:**

Precise identification of dermatophyte species significantly improves treatment and controls measures of dermatophytosis in human and animals. This study was designed to evaluate molecular tools effectiveness of the gene sequencing and DNA-based fragment polymorphism analysis for accurate identification and differentiation of closely- related dermatophyte species isolated from clinical cases of dermatophytosis and their antifungal susceptibility to the current antifungal agents.

**Materials and Methods:**

In this experimental study, a total of 95 skin samples were inoculated into mycobiotic agar for two weeks at 28˚C. Morphological characteristics of the isolated dermatophytes were evaluated. DNA was extracted from the fungal culture for amplification of topoisomerase II gene fragments and polymerase chain reaction (PCR) products were digested by Hinf I enzyme. Internal transcribed spacer (ITS) rDNA and *TEF-1α* regions of the all isolates were amplified using the primers of ITS1/4 and EF-DermF/EF-DermR, respectively.

**Results:**

Based on the morphological criteria, 24, 24, 24 and 23 isolates were identified as *T. rubrum, T. interdigitale, T. tonsurans* and *E. floccosum*, respectively. PCR-restriction fragment length polymorphism (RFLP) results provided identification pattern of the isolates for *T. rubrum* (19 isolates), *T. tonsurans* (28 isolates), *T. interdigitale* (26 isolates) and *E. floccosum* (22 isolates). Concatenated dataset results were similar in PCR-RFLP, except six *T. interdigitale* isolates belonging to *T. mentagrophytes*.

**Conclusion:**

Our results clearly indicated that conventional morphology and PCR-RFLP were not able to precisely identify all dermatophyte species and differentiation of closely related species like *T. interdigitale* and *T. mentagrophytes*, while ITS rDNA and *TEF-1α* gene sequence analyses provided accurate identification of all isolates at the genus and species level.

## Introduction

Dermatophytosis is a superficial fungal infection caused by dermatophytes, affecting nearly 20% of the population worldwide, as a public health problem (1, 2). Previous studies revealed a significant increase in dermatophyte infections (3, 4). Over 40 species of dermatophytes were assigned to three genera, including *Trichophyton, Epidermophyton* and Microsporum (2, 5). All three groups can infect humans via direct or indirect contact (5). Ordinarily, dermatophyte species like *T. interdigitale, T. rubrum, T. tonsurans* and *E. floccosum* are major etiologic agents of dermatophytosis in Iran (6-9). Communicating epidemiological statistics of these dermatophytes is greatly impeded, since taxonomic schemes are constantly changing. For example, it has recently been revealed that the previous *T. mentagrophytes* complex is composed of four new species: i. Zoophilic *T. Mentagrophytes* sensustricto, ii. Zoophilic *T. erinacei*, iii. *Trichophyton* anamorph of *A. benhamiae* (zoophilic), and iv. Zoophilic and anthropophilic strains of *T. interdigitale* (10, 11). Based on the latest classification, anthropophilic *T. mentagrophytes* should now be relabelled as *T. interdigitale* (12, 13). Regarding morphological similarity among the dermatophytes spp., epidemiology variation of dermatophytes and emerging new pathogens, it is necessary to identify isolates at the species level (3, 7, 8). 

Dermatophytosis is routinely identified by direct examination and culture (14). The phenotypic features depend on many variables such as the slow growth rate, temperature variation, prior therapy and production of spores (2, 7, 15). In addition, the clinical signs of dermatophytosis are often atypical in immunocompromised hosts (7). Moreover, routine procedures are either slow or nonspecific (6, 15, 16), and requires training of personnel and supervisory expertise (17, 18). Furthermore, phenotypic methods fail to closely discriminate the related species. Developing molecular methods provided more accurate and rapid results for differentiating species of dermatophytes. Polymerase chain reaction (PCR) and DNA fragments sequencing of the internal transcribed spacer (ITS) regions, 18S rDNA, translation elongation factor1-a (TEF-1α), restriction fragment length polymorphism analysis (RFLP), nested PCR, repetitive sequence PCR (rep-PCR), arbitrarily primed- PCR (AP-PCR) and real-time PCR are some examples of these methods (15, 17, 19-25). At present, sequence of the ITS region is considered as the gold standard for dermatophyte analyses (14, 26). *TEF-1α* gene was considered as an alternative to rDNA showing high level of variation rate among the species (25). The results obtained by previous studies suggest that PCR-RFLP assay is more efficient and convenient for fungal diagnosis. PCR-RFLP studies targeting the ITS rDNA have shown that it is a reliable method for identification of dermatophytes at the species level (27-29). It has been reported that DNA topoisomerase II gene is useful as a target for the study of different fungal species (26). Despite various studies about the significance of species identification in dermatophytes, to the best of our knowledge, limited data has been published on the precise differentiation of dermatophytes spp. by combination of the ITS and TEF1a sequences and topoisomerase II PCR-RFLP approach. The present study was evaluated the effectiveness of gene sequencing and DNA-based fragment polymorphism analysis molecular tools for accurate identification and differentiation of closely-related dermatophyte species isolated from clinical cases of dermatophytosis and their antifungal sensitivity to the current antifungal agents. 

## Materials and Methods

### Specimens and conventional assays 

In this experimental study, a total of 95 hair and skin samples, from patients suspected to dermatophytosis, were received for routine examination at Department of Mycology of Pasteur Institute (Iran). This study was approved by Ethical Committee of Pasteur Institute of Iran (Code No. IR.PII.REC.1397.021). Patients were informed of the procedure. Direct microscopy examination of the samples was performed using 10% potassium hydroxide and the samples were cultured on mycobiotic agar (Merck, Germany) plates to facilitate growth of the dermatophytes. The plates were incubated at 30˚C for 4 weeks. All fungal isolates were identified by analysis of the morphological characteristics (typical macro/microscopic characters of the colonies, and additional tests like hair perforation or urease tests). The dermatophyte strains including *T. tonsurans, T. interdigitale, E. floccosum* and *T. rubrum* were identified by morphological characterization. In addition, standard strains of *T. rubrum* (PFCC 51431), *T. mentagrophytes* (PTCC 5054), *T. tonsurans* (CBS 130924) and *E. floccosum* (CBS 767.73) were included in the study. The dermatophyte strains were then cultured and identified by analysis of the molecular methods. To validate standard strains at species level based on the latest classification, all strains were sequenced.

### Molecular identification and differentiation of dermatophyte species

#### DNA extraction

All clinical and standard strains were cultured on mycobiotic agar (Merck, Germany) and incubated at 28°C for two weeks. A fungal colony was cut from the agar plate with a scalpel, transferred to a mortar and grounded in liquid nitrogen. Then, using the phenol-chloroform-isoamyl alcohol chemicals, DNA was extracted according to Makimura et al. (26).

#### PCR-RFLP assay targeting the topoisomerase II 

The PCR was performed using a Taq DNA Polymerase Master Mix, with topoisomerase II primer (dPsD2)

dDPF2: 5´-GTYTGGAAYAAYGGYCGYGGTATTCC-3´ and
dDPR2: 5´-AAVCCGCGGAACCAKGGCTTCATKGG-3´.

PCR program was performed by the following cycle conditions: an initial denaturation at 95°C for 5 minutes, followed by 30 cycles of 95°C for 30 seconds, 63°C for 15 seconds, and 72°C for 120 seconds, followed by a final extension at 72°C for 5 minutes (19). The PCR products with ~2380 bp length were purified using a Min Elute PCR Purification kit (Qiagen, USA). 

#### Restriction fragment length polymorphism analysis of the amplified topoisomerase II

Digestion of all reactions were performed in 15 µl mixturevolume containing 2 µl of 10× buffer (Fermentas, USA), 2 µlof each enzyme, 10 µl purified PCR products and sufficientamount of ultrapure water to approach final volume. Digestionwas performed using Hinf I reaction enzyme (Fermentas, USA) at 37°C for 8-10 hours (18). PCR amplicons andrestriction enzyme digestion products were loaded in 2.5% (w/v) agarose gels in the presence of a GelRed stain (BiotiumInc., USA) (0.5 µg/ml), while a 100 bp DNA molecular sizemarker (Fermentas, USA) was used, and the sample were run at 90 V/Cm for 90 minutes. 

#### Internal transcribed spacer and *TEF-1α* region amplifications by PCR 

For each sample, the TEF-1α and ITS regions were amplified using the specific primers 

EF-Derm

F: 5´-CACATTAACTTGGTCGTTATCG-3´ and R: 5´-CATCCTTGGAGATACCAGC-3´, as well as ITS1: 5´- TCCGTAGGTGAACCTGCGG-3´ and ITS4: 5´-TCCTCCGCTTATTGATATGC-3´. 

The reaction PCRs were consisted of initially denaturation at 95°C for 5 minutes, followed by 30 cycles of 94°C for 30 seconds, 58°C for 30 seconds and 72°C or 45 seconds, followed by a final extension step at 72°C for 5 minutes (25, 30). 

#### Sequencing

Purified PCR product was sequenced using the ABI PRISM BigDye Terminator Cycle Sequencing Ready Reaction Kit (Applied Biosystems, USA). 

#### Phylogenetic analysis

The best-fit model of molecular evolution was estimated in jModelTest 2.1.10 (31). Sequences of the two loci of each isolate were combined for phylogenetic analyses with PAUPversion 4.0b109 (32). The program MrBayes version 3.2 (33), run on the CIPRES Science Gateway (34). Two simultaneousanalyses with eight Metropolis-coupled Markov chain Monte Carlo (MCMC) chains with incremental heatingof 0.2 were run for 20 million generations, sampled every1000 generations. We verified the convergence of parameterestimates and the effective sample sizes were > 200bp for all parameters using Tracer version 1.6 (35). 

#### Antifungal drug susceptibility testing

Terbinafine, griseofulvin and ketoconazole (Sigma- Aldrich, USA) were prepared in dimethyl sulfoxide (DMSO). Final concentration of drugs, fungal spore suspensions, wereprepared in the standard RPMI 1640 medium (Sigma-Aldrich, USA) buffered to pH=7.0 with 0.165 mol/l 3-(N-morpholino) propanesulfonic acid (MOPS) with L-glutamine (Sigma- Aldrich, USA), with no bicarbonate, in 96-well round bottommicroplates according to CLSI M38-A2 broth microdilutionprotocol (36). All tests were performed in triplicate. Theinoculated microplates were incubated at 35°C and visuallyassessed for fungal growth after four days incubation. Theminimum inhibitory concentration (MIC) was defined as thepoint at which the growth of dermatophyte was inhibited by80% for three antifungals, in comparison with the control. T. rubrum (PTCC 5143) and C. parapsilosis (ATCC 22019) were used as quality controls. MIC range, as geometric mean, was provided for all of the tested isolates. 

### Results

#### Identification of dermatophyte species using conventional assays

Morphological identification of isolated dermatophytes by using a combination of macroscopic (colony morphology, texture and color) and microscopic (hyphae structure, shape of macroconidia and microconidia) features showed that all the isolates were distributed in four species including *T. rubrum* (n=24), *T. tonsurans* (n=24), *T. interdigitale* (n=24) and *E. floccosum* (n=23). 

#### Identification of the dermatophyte species by PCR- RFLP 

The genomic DNAs were amplified with dPsD2 and generated a 2380 bp band. Amplification profile of theproducts were also identified for all 99 strains. The sizeswere expected from the region amplified by dPsD2 andthe restriction enzyme digestion with Hinf I (Table 1) wasobtained from the website NEB cutter (http://tools.neb.com/ NEBcutter). The PCR products were digested with Hinf I. Thebanding patterns obtained by the PCR-RFLP are shown inFigure 1. After amplification of genomic DNAs using dPsD2, the expected size was generated for all isolates. Differencesbetween the fragments with less than 20 bp differenceswas not showed; therefore, there was overlap in the bands70 bp and 67 bp in *T. tonsurans* as well as two distinctive bands (255 bp and 260 bp) and (178 bp and 186bp) in *E. floccosum*. All specimens were identified at the species level by the unique banding pattern specified to each species. All of the banding patterns for each species were coincided with its standard strains. PCR-RFLP results provided identification pattern of the isolates as *T. rubrum* (n=19), *T. tonsurans* (n=28), *T. interdigitale* (n=26) and *E. floccosum* (n=22). 

**Table 1:** The expected sizes of DNA fragments generated by enzymatic digestion of *Hinf I*


Dermatophyte species (no.)	DNA fragment (bp)

*T. interdigitale* (27)	1209, 482, 233, 166, 137, 95, 58
*T. rubrum* (20)	1267, 482, 370, 262
*T. tonsurans* (29)	1209, 482, 233, 166, 95, 70, 67, 58
*E. floccosum* (23)	954, 482, 260, 255, 186, 178, 58


**Fig.1:**
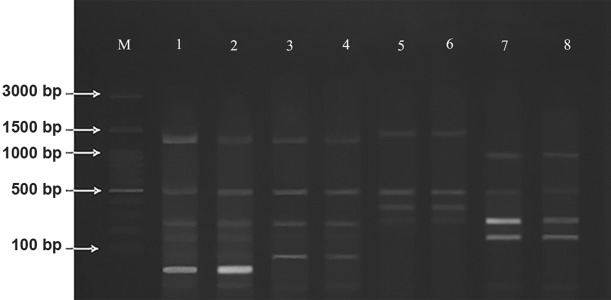
Polymerase chain reaction-restriction fragment length polymorphism (PCR-RFLP) electrophoretic patterns of dermatophytes species by amplification of topoisomerase II gene and digestion of the Hinf I enzyme. Lane M; 100 bp DNA ladder, Lane 1; *T. tonsurans,* Lane 2; *T. tonsurans *(CBS 130924), Lane 3; *T. interdigitale*, Lane 4; *T. mentagrophytes,* Lane 5; *T. rubrum,* Lane 6; *T. rubrum* (PFCC 51431), Lane 7; *E. floccosum*, and Lane 8; *E. floccosum *(CBS 767.73).

#### Identification of dermatophyte species by PCR sequencing

In the present study, all dermatophytes spp. (clinical and standard strains) were identified based on ITS sequencing. ITS and *TEF-1α* sequences of the isolates were aligned using ClustalW as implemented in MEGA7.0.21 software and edited manually to improve the alignment accuracy. The query sequences were paired with those in the GenBank database, using the Blastn analysis. On the basis of sequencing results, the dermatophyte isolates included *T. rubrum *(n=20), *T. tonsurans* (n=29), *T. interdigitale* (n=21), *T. mentagrophytes* (n=6) and *E. floccosum* (n=23). The *ITS/TEF-1α* sequence interpretations revealed that six isolates were identified as *T. interdigitale*, in contrary of PCR-RFLP results that showed *T. interdigitale* and *T. mentagrophytes* is categorized in same species. 

**Fig.2:**
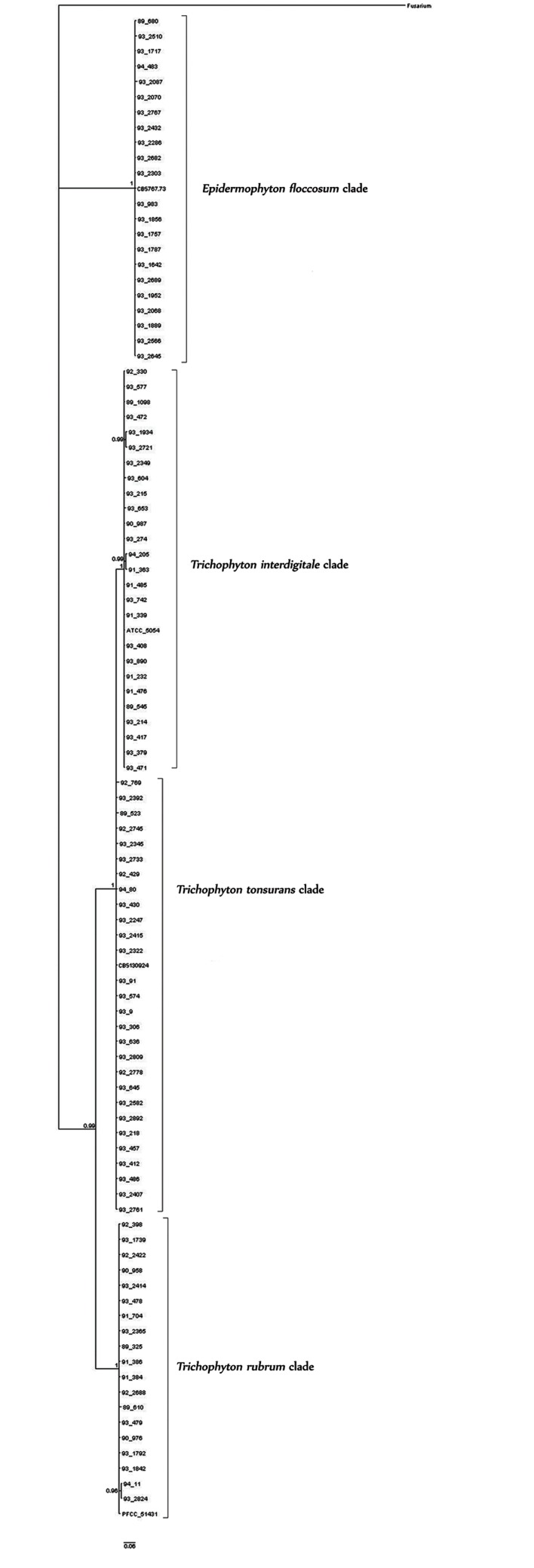
Bayesian tree based on the combined dataset. Phylogenetic analysis of the combined dataset with TIM2+G model of the 95 clinical isolates, four standard strains and Fusarium, as the out-group. Posterior probabilities more than 60% are given for the appropriate clades.

A consensus tree belonging to the ITS and *TEF-1α* fragment was constructed for all species discussed in this study (Fig .2). Four clades were distinguishable. Furthermore, *T. mentagrophytes* and *T. interdigitale* were placed in the distinctive clusters. The dendrogram describes the relationships between all of the studied isolates. Isolates belonging to any species were clustered with a high support (more than 60%) in separate clades. 

#### Molecular versus conventional method of species identification

Table 2 shows the results of conventional method of species identification and PCR-RFLP. The results of identification of dermatophyte spp. using PCR-RFLP were confirmed by sequencing of the ITS and *TEF-1α* regions. PCR-RFLP showed an increase in the identification rate compared to the conventional method. Analysis dataset of ITS and *TEF-1α* indicated that six isolates belonged to* T. mentagrophytes* and 21 isolates belonged *T. interdigitale,* while topoisomerase II PCR-RFLP failed to discriminate them. Interestingly, a complete overlap was observed between both methods in the case of remaining isolates.

Sensitivity of the molecular method was more than sensitivity of the conventional method. The results indicated that 86.4% of dermatophyte spp. identified by the conventional method was also confirmed by the molecular method. The specificity and sensitivity of sequencing method were found to be approximately 100%. Utilizing molecular method demonstrated that six out of the 24 isolates, identified as *T. rubrum* by conventional method, belonged to another genus and species including, *T. interdigitale* (n=4), *E. floccosum* (n=1) and *T. tonsurans* (n=1), using molecular method. Among the 24 strains identified as *T. interdigitale* by morphological examination, four strains had also been recognized as *T. tonsurans* (n=2), *E. floccosum* (n=1), and *T. rubrum* (n=1) by molecular methods. Three of 23 isolates which were identified as *E. floccosum*, by morphological examination were re-identified and confirmed as *T. interdigitale* (n=2) and *T. tonsurans* (n=1) by molecular characteristics. 

#### Antifungal drug sensitivity of dermatophyte isolates 

The MIC range and geometric mean were obtained for the all dermatophyte species (Table 3). A significant sensitivity to terbinafine was reported in *T. tonsurans*. The most sensitive and resistant species to griseofulvin were *T. interdigitale* and *E. floccosum*, respectively. Terbinafine and griseofulvin had the lowest and the highest geometric mean MICs, which were respectively 0.01 and 1.64 µg/ml for *T. interdigitale* and *E. floccosum*. Terbinafine was the most effective antifungal drug against all dermatophyte species. 

**Table 2:** Identification of dermatophytes based on morphological andmolecular methods


Dermatophytes spp.	Morphological identification			
	*T. interdigitale*	*T. rubrum*	*T. tonsurans*	*E. floccosum*

*T. interdigitale* (n=26)	20	4	-	2
*T. rubrum* (n=19)	1	18	-	-
*T. tonsurans* (n=28)	2	1	24	1
*E. floccosum* (n=22)	1	1	-	20
Total	24	24	24	23


**Table 3:** In vitro antifungal susceptibility of dermatophytes against three antifungal agents


Dermatophyte species	Antifungal drug	MIC Range	G mean

*T. interdigitale* (n=27)	Terbinafine	0.003-0.125	0.01
	Griseofulvin	0.03-64	0.41
	Ketoconazole	0.03-4	0.32
*T. rubrum* (n=20)	Terbinafine	0.003->32	0.04
	Griseofulvin	0.06-64	0.66
	Ketoconazole	0.06-8	0.28
*T. tonsurans* (n=29)	Terbinafine	0.003->32	0.01
	Griseofulvin	0.03-64	0.46
	Ketoconazole	0.03-2	0.16
*E. floccosum* (n=23)	Terbinafine	0.003-1	0.02
	Griseofulvin	0.03-64	1.64
	Ketoconazole	0.03-2	0.11


MIC; Minimum inhibitory concentration (µg/ml) and G mean; Geometric mean MIC.

### Discussion

As earlier mentioned, there was high similarity within dermatophytes species. In the present study, the obtained results using DNA sequencing method,to identify common dermatophyte spp., had 100% accuracy. In this study, about 20% of the dermatophyte spp. identified by the conventional method was not correct and molecular analysis showed in fact that 16.6% (n=4 out of the 24) strains identified as T. rubrum by morphological examination were *T. interdigitale*. Due to the similarity in the morphological characters of *T.mentagrophytes, T. rubrum *and *T. interdigitale*, their differentiation was remained challenge (37, 38). 

Interestingly, all 24 isolates, identified as *T. tonsurans* by morphological examination, were confirmed by molecular method. This highlighted the rare production of macroconidia by *T. tonsurans* leading to right identification at the phenotypic level. On the other hand, the *T. tonsurans* isolates with macroconidia were misidentified with *T. rubrum* and *T. interdigitale*. The topoisomerase-RFLP not only differentiated *T. rubrum* from *T. interdigitale,* but also it was a useful method for the differentiation of *T. interdigitale* from *T. tonsurans* by forming the unique bonding pattern for each species. The result was similar to what was reported by Kamiya et al. (7), showing that six dermatophyte spp. were specifically identified by the topoisomerase-RFLP. It should also be noted that similar to the study of Kanbe et al. (27), dermatophyte spp. were amplified by primer dPsD2. This was used for the common species identification of Trichophyton, Microsporum and Epidermophyton. The study conducted by Mochizuki et al. (29) demonstrated that ITS-RFLP of dermatophyte spp. was a reliable method for rapid identification of this fungus. Besides that, *TEF-1α* gene was considered as an alternative to rDNA that shows a high level of variation rate among the species. Findings obtained by Mirhendi et al. (25) are in accordance with our results. Result of the present study indicated that ITS/*TEF-1α* combination is a valuable approach to omit possible misidentification among the closely related species. 

To correctly identify dermatophytes based on morphological characteristics, 2-4 weeks are required, while application of the molecular method showed that DNA derived from a fresh colony -cultured for five days- is suitable for identification these fungi. It was shown that some closely related species like *T. equinum *and *T. tonsurans* as well as *M. canis* and *M. ferrugineum*, showing no pattern difference in the ITS-RFLP (37), should be investigated using topoisomerase-RFLP. Although the topoisomerase-RFLP was rapid, stable and reproducible for the common dermatophytes spp., it is not a convenient tool for distinguishing between *T. interdigitale* and *T. mentagrophytes*. 

## Conclusion

Precise identification of dermatophyte species significantly improves treatment and control of dermatophytosis in human and animals. Our results clearly indicated that conventional morphology and PCRRFLP are not able to precisely identify all dermatophyte species and differentiate the closely related species like *T. interdigitale* and *T. mentagrophytes*, while ITS rDNA and TEF-1α gene sequence analyses provided an accurate identification for the all isolates at the genus and species level. Thus, concurrent sequence analysis of these genomic regions is very useful to confirm identity of dermatophyte species identified by routine morphology. It also enables clinicians for recommending effective treatment and control strategies to overcome various clinical types of dermatophytosis, especially chronic infection, which are antifungal drug resistance and quite difficult to treat.
